# Tocotrienol-Rich Fraction Ameliorates Antioxidant Defense Mechanisms and Improves Replicative Senescence-Associated Oxidative Stress in Human Myoblasts

**DOI:** 10.1155/2017/3868305

**Published:** 2017-01-24

**Authors:** Shy Cian Khor, Wan Zurinah Wan Ngah, Yasmin Anum Mohd Yusof, Norwahidah Abdul Karim, Suzana Makpol

**Affiliations:** Department of Biochemistry, Faculty of Medicine, Level 17, Preclinical Building, Universiti Kebangsaan Malaysia Medical Centre (UKMMC), Jalan Yaacob Latif, Bandar Tun Razak, 56000 Cheras, Kuala Lumpur, Malaysia

## Abstract

During aging, oxidative stress affects the normal function of satellite cells, with consequent regeneration defects that lead to sarcopenia. This study aimed to evaluate tocotrienol-rich fraction (TRF) modulation in reestablishing the oxidative status of myoblasts during replicative senescence and to compare the effects of TRF with other antioxidants (*α*-tocopherol (ATF) and* N*-acetyl-cysteine (NAC)). Primary human myoblasts were cultured to young, presenescent, and senescent phases. The cells were treated with antioxidants for 24 h, followed by the assessment of free radical generation, lipid peroxidation, antioxidant enzyme mRNA expression and activities, and the ratio of reduced to oxidized glutathione. Our data showed that replicative senescence increased reactive oxygen species (ROS) generation and lipid peroxidation in myoblasts. Treatment with TRF significantly diminished ROS production and decreased lipid peroxidation in senescent myoblasts. Moreover, the gene expression of superoxide dismutase* (SOD2)*, catalase* (CAT),* and glutathione peroxidase* (GPX1)* was modulated by TRF treatment, with increased activity of superoxide dismutase and catalase and reduced glutathione peroxidase in senescent myoblasts. In comparison to ATF and NAC, TRF was more efficient in heightening the antioxidant capacity and reducing free radical insults. These results suggested that TRF is able to ameliorate antioxidant defense mechanisms and improves replicative senescence-associated oxidative stress in myoblasts.

## 1. Introduction

Adult skeletal muscle contains a subpopulation of cells that readily proliferate and differentiate when required to maintain the structure and function of skeletal muscle [1]. These cells were first identified by Mauro in 1961 as quiescent cells located between the basal lamina and sarcolemma of myofibers, known as satellite cells [2]. Human satellite cells can be isolated and cultured in vitro with a limited proliferative capacity depending on the donor age. Proliferating satellite cells are known as myoblasts [3]. The proliferative lifespan of myoblasts remains stable during adulthood but decreases from infants to adolescents, and the cells ultimately reach replicative senescent [4].

During aging, a progressive loss of muscle mass and strength is observed, and this phenomenon is known as sarcopenia. Although the underlying mechanism is still uncertain, sarcopenia is believed to be the result of certain intrinsic or extrinsic factors, such as immobilization, chronic diseases, changes in hormone, and proinflammatory factors, as well as nutritional status in older adults [5]. Additionally, the accumulation of reactive oxygen species (ROS) has been suggested to play a vital role in this age-related muscle atrophy [6]. Redox imbalance observed in senescent satellite cells can be attributed to elevated ROS production or an impaired endogenous antioxidant defense system, leading to oxidative damage [7, 8]. The vulnerability of proliferating myoblasts to oxidative damage will affect muscle regeneration and contributes to the development of sarcopenia, suggesting that oxidative stress, satellite cells, and sarcopenia are interrelated [6, 7].

Oxidative stress in aged skeletal muscle can cause oxidative damage in cells, manifested as damaged DNA, lipid peroxidation, and protein carbonylation [9, 10]. In muscle fibers, free radicals can be produced intrinsically by mitochondria and regulate fundamental signaling pathways in skeletal muscle. The presence of reactive oxygen species (ROS) or reactive nitrogen species (RNS) can be counteracted by the antioxidant defense system, which includes antioxidant enzymes, vitamins, and glutathione, resulting in sustained redox balance [9]. If the antioxidant defense is overwhelmed by excess ROS or RNS, oxidative stress occurs which leads to muscle injury [8, 10].

In addition to the existing oxidative stress during aging, insufficient antioxidant intake among the elderly can contribute to the occurrence of sarcopenia [11]. Low antioxidant levels in older individuals were associated with poor muscle strength and low physical performance and can cause frailty in the elderly [12, 13]. An in vivo study demonstrated that vitamin E deficiency caused poor muscle performance and accelerated aging development [14]. Hence, introducing antioxidants such as vitamin E could be a relevant strategy to delay sarcopenia progression; however, more studies are needed [15].

Vitamin E is a lipid-soluble vitamin with two subclasses, tocopherols and tocotrienols [16]. A previous study reported that *α*-tocopherol was able to repair laser-induced disrupted myoblast membranes, indicating a therapeutic effect for vitamin E in the muscle [17]. However, the less-explored subtype of vitamin E is the class of tocotrienols. Similar to *α*-tocopherol, a potential therapeutic effect of tocotrienol-rich fraction (TRF) was proposed owing to its reversal effect on stress-induced presenescence (SIPS) model of myoblasts [18]. In our laboratory, we also found that TRF was superior to *α*-tocopherol in ameliorating replicative senescence-related aberration and promoting myogenic differentiation [19]. Thus, it would be of interest to elucidate the effects of tocotrienols on the dynamics of oxidative status in senescent myoblasts. Therefore, the aims of this study were to investigate the effects of tocotrienol-rich fraction (TRF) in reestablishing oxidative status during replicative senescence of myoblasts and to compare these effects with other antioxidants, such as *α*-tocopherol (ATF) and* N*-acetyl-cysteine (NAC), in young, presenescent, and senescent myoblasts followed by the measurement of cell viability and apoptosis as the final outcomes of antioxidant treatment.

## 2. Materials and Methods

### 2.1. Cell Culture

Human Skeletal Muscle Myoblasts (HSMM) were purchased from Lonza (Walkersville, MD, USA). Briefly, myoblasts were cultured in Skeletal Muscle Basal Medium (SkBM) that was supplemented with human epidermal growth factor, fetal bovine serum, dexamethasone, L-glutamine, and gentamicin sulfate/amphotericin B (Lonza, Walkersville, MD USA). Cells were cultivated at 37°C in a humid atmosphere containing 5% CO_2_. The myoblasts then underwent serial passaging. The number of divisions was calculated for each passage using the formula ln⁡(*N*/*n*)/ln⁡2, where* N *is the number of cells at the harvest stage and* n* is the number of cells at the seeding stage [20]. When cells reached replicative senescence, they were unable to proliferate within 10 days in culture. Myoblasts were divided into 3 different stages, young (<15 cell divisions), presenescent (18-19 cell divisions), and senescent (>20 cell divisions), based on their decreasing proliferative capacity which was represented by hyperbolic proliferative lifespan curve and diminishing percentage of BrdU incorporation. The presence of senescent cells was confirmed by SA-*β*-gal staining [19].

### 2.2. Antioxidants

Tocotrienol-rich fraction (TRF) was purchased from Sime Darby Sdn. Bhd., Selangor, Malaysia (TRF Gold TRI E 70), while alpha-tocopherol (ATF) was a gift from the Malaysian Palm Oil Board (MPOB) (Selangor, Malaysia). Both vitamin E subclasses are palm oil-derived. TRF consists of *α*-tocotrienol (ATT; 26.89%), *β*-tocotrienol (BTT; 3.64%), *γ*-tocotrienol (GTT; 31.66%), *δ*-tocotrienol (DTT; 13.66%), and *α*-tocopherol (ATF; 24.15%). Briefly, stock solutions of TRF were freshly prepared in 100% ethanol (1 : 1) and kept at −20°C for no longer than one month. A similar process was performed for ATF. TRF and ATF were then incubated overnight with fetal bovine serum at 37°C before use. Then, both TRF and ATF were diluted in culture medium and used at a final concentration of 50 *μ*g/mL [19].* N*-Acetyl-cysteine (NAC) was purchased from Sigma-Aldrich (St Louis, USA). NAC was freshly prepared in culture medium to the desired final concentration. A dosage of 1.0 mg/mL NAC was used for subsequent experiments, which was determined using a cell viability assay (Supplemental 1A in Supplementary Material available online at https://doi.org/10.1155/2017/3868305). A dosage of 25 *μ*g/mL of TRF or ATF with 1.0 mg/mL NAC was used for combined treatment of TRF and NAC (TRF + NAC) and combination of ATF and NAC (ATF + NAC) (Supplemental 1B and C).

### 2.3. Cell Viability

The optimal concentrations of NAC, the combination of TRF and NAC (TRF + NAC), and the combination of ATF and NAC (ATF + NAC) were determined using a cell viability assay (Supplemental 1). The effects of H_2_O_2_ and antioxidants were also determined using cell viability assay. A CellTiter 96® Aqueous Nonradioactive Cell Proliferation Assay (MTS; Promega, Madison, USA) was used according to the manufacturer's instructions. Cells were incubated with several concentrations of antioxidants for 24 h and 45 min for H_2_O_2_. After that, the treatments were replaced by MTS for another 2 h of incubation. The absorbance of MTS formazan was measured at 490 nm with a microtiter plate reader (VersaMax Molecular Devices, USA). The optimum dosage of treatments was used for subsequent experiments.

### 2.4. Analysis of Cell Morphology

In brief, cells were plated and fixed with cold ethanol in *μ*-Slide 8 wells (ibidi, Martinsried, Germany) followed by incubation with an anti-Desmin antibody (dilution: 1 : 50) (D33; Dako, Produktionsvej, Denmark) and Alexa Fluor 488 goat anti-mouse (dilution: 1 : 500) (Molecular Probes, Eugene, OR, USA). Nuclei were visualized using Hoechst 33342 (Molecular Probes, Eugene, OR, USA). Slides were then observed under a Leica TCS SP5 II Confocal Laser Scanning Microscope (Leica Microsystems, Wetzlar, Germany).

### 2.5. Senescence-Associated *β*-Galactosidase (SA *β*-gal) Staining

SA *β*-gal was evaluated using a Senescent Cell Histochemical Staining Kit (Sigma-Aldrich, St. Louis, Missouri, USA). Cells were stained according to the manufacturer's instructions. After the cells were incubated in staining solution for 8 h at 37°C in the absence of CO_2_, at least 100 cells were observed under the microscope, and the percentage of blue stained cells was calculated.

### 2.6. Assessment of the Intracellular Production of Reactive Oxygen Species (ROS)

ROS generation in myoblasts was determined using two dyes, dihydroethidium (DHE) and 5-(and-6)-carboxy-2′,7′-dichlorodihydrofluorescein diacetate (carboxy-H_2_DCFDA) (Molecular Probes, Eugene, OR, USA). The fluorescence of DHE indicated oxidation by superoxide anions, while carboxy-H_2_DCFDA is oxidized by hydrogen peroxide (H_2_O_2_), peroxynitrite, or hydroxyl radicals. Superoxide anions may contribute to carboxy-H_2_DCFDA oxidation albeit to a lesser degree. Briefly, myoblasts were incubated in 20 *μ*M DHE and 40 *μ*M carboxy-H_2_DCFDA for 45 min. After that, cells were washed with PBS and recovered in medium for 30 min. Then, we measured the intensity of the fluorescence using a flow cytometer (CytoFLEX, Beckman Coulter Pasadena, CA, USA) with the 585/42 bandpass and 525/40 bandpass channels. The percentage of cells that was positively labeled with a particular dye was reported independently. For observation, the same staining protocol was applied, followed by visualization of the nuclei using Hoechst 33342 (Molecular Probes, Eugene, OR, USA). Cells were then observed under a fluorescence microscope (EVOS FL digital inverted microscope, Thermo Fisher Scientific, USA).

### 2.7. Assessment of Lipid Peroxidation

Lipid peroxidation in myoblasts was measured using a dye, C11-BODIPY® (581/591) (Molecular Probes, Eugene, OR, USA), which is a lipid peroxidation sensor that can shift from red to green fluorescence emission upon oxidation of the polyunsaturated butadienyl segment of the fluorophore. Briefly, myoblasts were incubated in 10 *μ*M C11-BODIPY for 30 min. After that, cells were washed with PBS, trypsinized, and reconstituted in PBS and the oxidized BODIPY was quantitated using a flow cytometer (CytoFLEX, Beckman Coulter Pasadena, CA, USA) with the 525/40 bandpass channel while reduced BODIPY was quantitated with 585/42 bandpass channel. The percentage of cells that was positively labeled with either oxidized or reduced BODIPY was obtained and the ratio of these percentages (oxidized/reduced BODIPY) was reported. For observation, the same staining protocol was applied, followed by visualization of the nuclei using Hoechst 33342 (Molecular Probes, Eugene, OR, USA). Cells were then observed under a fluorescent microscope (EVOS FL digital inverted microscope, Thermo Fisher Scientific, USA).

### 2.8. Cellular Uptake of Vitamin E

Vitamin E extraction was performed according to the protocol by Mazlan et al. [22]. After trypsinizing and counting the cells, 50 mg/mL butylated hydroxytoluene (BHT) was added to stop autooxidation. The hexane layer of supernatant was then collected, vacuum-dried, and stored at −80°C before analysis with HPLC. A total of 100 *μ*L hexane was added before further dilution with hexane for HPLC analysis. The uptake of vitamin E was analyzed using an HPLC fluorescence detector (Ex/Em: 294 nm/330 nm) (RF-10A, Shimadzu, Japan). A TRF standard was used, and the concentrations of *α*-tocopherol (ATF), *α*-tocotrienol (ATT), *β*-tocotrienol (BTT), *γ*-tocotrienol (GTT), and *δ*-tocotrienol (DTT) uptake in cells were calculated in *μ*g/mL per million cells.

### 2.9. Determination of Antioxidant Enzymes at the Transcriptional Level

Total RNA was extracted using TRI reagent and polyacryl carrier (Molecular Research Center Inc., Ohio, USA). For gene expression determination, quantitative real-time RT-PCR (qRT-PCR) was used. The expression of* SOD1*,* SOD2*,* CAT,* and* GPX1* mRNA was quantitatively analyzed using KAPA SYBR FAST One-Step qPCR kit (Kapa Biosystems, Boston, Massachusetts, USA). For RT-PCR, 400 nM of each primer was used, and the primer sequences are shown in Table 1 [21]. The master mix was prepared, and PCR reactions were carried out in a Bio-Rad iQ5 Cycler (Hercules, CA, USA). The program included cDNA synthesis for 5 min at 42°C; predenaturation for 4 min at 95°C; and PCR amplification for 40 cycles of 3 sec at 95°C and 20 sec at 60°C. These reactions were followed by a melt curve analysis of each targeted gene. The melt curve analysis of each pair of primers and agarose gel electrophoresis that was performed on the PCR products were used to determine the primer specificity (Supplemental 2). The expression level of each targeted gene was normalized to that of glyceraldehyde 3-phosphate dehydrogenase (*GAPDH*). The relative expression value (REV) was calculated using the 2^−ΔΔCt^ method of relative quantification and the following equation:

(1) $$\begin{array}{c} \text{REV}=2^{\text{Ct value of GAPDH}-\text{Ct value of the gene of interest}}. \end{array}$$

### 2.10. Activities of Antioxidant Enzymes

The activities of three antioxidant enzymes, superoxide dismutase (Sod), catalase (Cat), and glutathione peroxidase (Gpx), were determined. These enzymes were extracted in PBS by sonication following the 24-hour treatments.

A Sod assay was performed according to Beyer Jr. and Fridovich [23]. In brief, substrate solution was freshly prepared by mixing L-methionine, nitro blue tetrazolium (NBT), 1% Triton-X, and PBS, pH 7.8 (Sigma, St Louis, USA). Then, 20 *μ*L of enzyme extract and 10 *μ*L of 4 mg/100 mL riboflavin (Sigma, St Louis, USA) were added before incubation under 20-W Sylvania GroLux lamps in a cupboard for 7 min. Absorbance was measured using a UV/VIS spectrophotometer (Shimadzu, Kyoto, Japan) at 560 nm. Sod specific activity was expressed in mU/mg of protein.

A Cat assay was carried out using the method described by Aebi [24]. Enzyme extract was added to a quartz cuvette. The reaction was started by adding 30 mM H_2_O_2_ (Merck, Darmstadt, German), and absorbance was measured kinetically for 30 seconds using a UV/VIS spectrophotometer (Shimadzu, Kyoto, Japan) at 240 nm. Cat-specific activity was expressed in mU/mg of protein.

A Gpx assay was carried out according to Paglia and Valentine [25]. Substrate solution was freshly prepared by mixing reduced glutathione, PBS at pH 7.0, sodium azide, the reduced form of nicotinamide adenine dinucleotide phosphate (NADPH), and glutathione reductase (1 U/mL) (Sigma, St Louis, USA). Enzyme extract was added to the substrate solution, and the reaction was started by adding H_2_O_2_ (Merck, Darmstadt, German). The conversion of NADPH to NADP^+^ was measured kinetically using a UV/VIS spectrophotometer (Shimadzu, Kyoto, Japan) at 340 nm for 5 min. Gpx-specific activity was expressed in mU/mg of protein.

The total protein was extracted using lysis buffer, and its concentration was determined using a Bio-Rad Protein Assay (Hercules, CA, USA) at 595 nm with a microtiter plate reader (VersaMax Molecular Devices, USA). The protein concentration was used to normalize the enzyme activity.

### 2.11. Measurement of Reduced to Oxidized Glutathione (GSH/GSSG) Ratio

The GSH/GSSG ratio was determined using a GSH/GSSG-Glo™ Assay Kit (Promega, Madison, WI), which is based on the firefly luciferase reaction. Based on the manufacturer's instructions, both the GSH and GSSG luminescent reaction schemes were performed and measured using a microplate reader with an integration time of 1 s/well (Infinite® 200, Tecan, USA). The ratio was calculated directly from Net RLU as stated in the technical manual.

### 2.12. Assessment of Apoptotic Events

Apoptosis profiles were determined using an Annexin V-FITC Apoptosis Detection Kit II (BD Pharmingen, CA, USA) according to the manufacturer's instructions. Two dyes were used, Annexin V-FITC, which was detected by FL1, and propidium iodide (PI), which was detected by FL3. The cells were analyzed with a FACS Calibur flow cytometer (Becton Dickinson, CA, USA). The percentage of cells that were negatively stained with both dyes (FITC^−ve^/PI^−ve^) was reported as viable cells, while percentage of cells that were positively stained with Annexin V-FITC only (FITC^+ve^/PI^−ve^) or both Annexin V-FITC and PI (FITC^+ve^/PI^+ve^) was reported as early and late apoptotic cells, respectively.

### 2.13. Statistical Analysis

Statistical analyses were performed using SPSS 22.0 software (IBM, NY, USA). All of the data are reported as the means ± standard deviation (SD) from at least three replicates. For all of the tests, *p* < 0.05 was considered statistically significant. To determine the significance between two treatment groups, comparisons were made using an independent *t*-test, while ANOVA was used to analyze multiple groups, followed by a post hoc Tukey's HSD or LSD (if equal variance was assumed) and Dunnett's T3 (if equal variance was not assumed) tests.

## 3. Results

### 3.1. Effects of Antioxidants on Senescent Myoblasts

**Figure 1:**
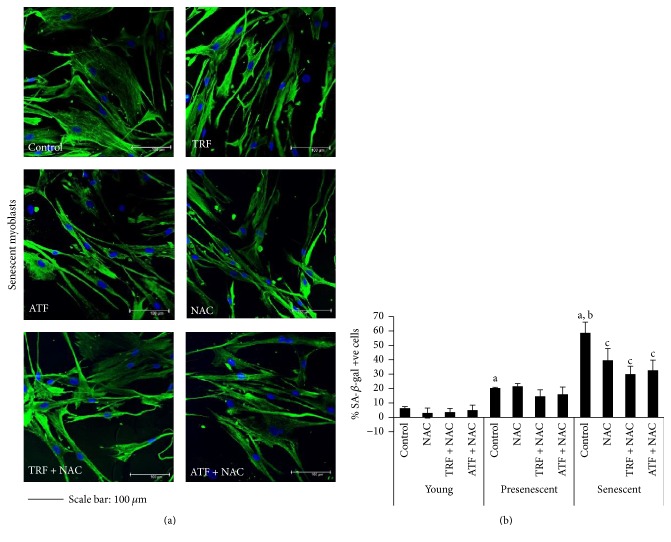
Protective effects of NAC alone and in combination with TRF and ATF on senescent myoblasts. (a) More spindle-shaped cells observed in senescent myoblasts after NAC, TRF + NAC, and ATF + NAC treatment, which were similar to the effect of TRF and ATF treatment alone on senescent myoblasts. Scale bar, 100 *μ*m. (b) The percentage of SA-*β*-gal positive cells decreased in all treatment groups. ^a^Denoting *p* < 0.05, significantly different compared to the young control, ^b^*p* < 0.05, significantly different compared to the presenescent control, and ^c^*p* < 0.05, significantly different compared to the senescent control. Data are presented as the mean ± SD, *n* = 3.

To determine whether antioxidant properties alone are sufficient to ameliorate senescent myoblasts, in our study we tested a synthetic antioxidant,* N*-acetyl-cysteine (NAC), and its combination with TRF and ATF, besides treatment with TRF or ATF alone. Previously, the beneficial effects of TRF or ATF on senescent myoblasts were reported, where a concentration of 50 *μ*g/mL TRF or ATF alone was able to increase cell viability, improve cellular morphology (more spindle-shaped cells were observed), and decrease the total of SA-*β*-gal staining positive cells during replicative senescence [19]. In the present study, we found that NAC was not toxic to cells up to the highest concentration used with a 24-hour incubation (Supplemental 1A). Thus, 1.0 mg/mL of NAC was used in the subsequent experiments. Different concentrations of TRF or ATF in combination with NAC were tested using cell viability assay (Supplemental 1B and 1C) in which a concentration of 25 *μ*g/mL TRF or ATF was used with 1.0 mg/mL NAC in the subsequent experiment. NAC alone and in combination with TRF (TRF + NAC) or ATF (ATF + NAC) significantly improved the cellular morphology of senescent myoblasts (cells became spindle-shaped) and reduced the percentage of cells positive for a senescence biomarker (SA-*β*-gal staining) (Figures 1(a) and 1(b)).

### 3.2. Effects of Antioxidants on ROS Generation during Replicative Senescence

**Figure 2:**
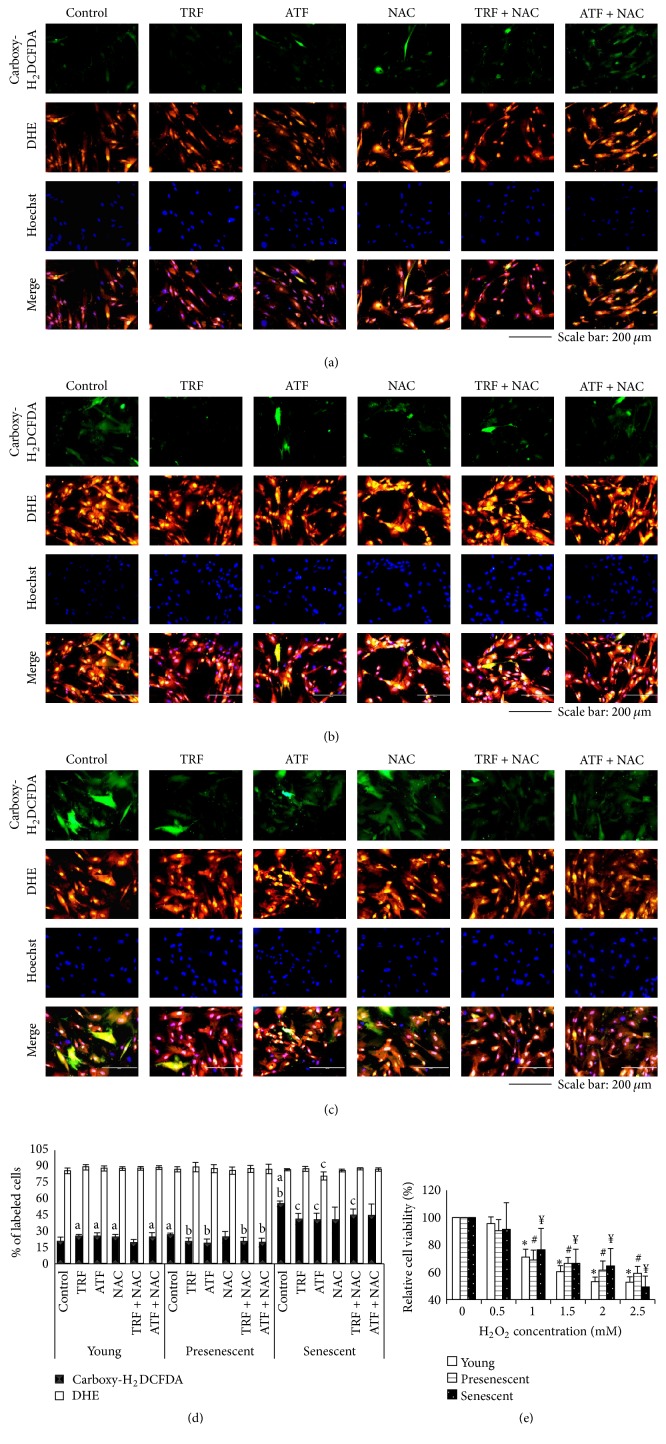
Effects of antioxidants treatment on the presence of ROS in myoblast cells. (a) Young, (b) presenescent, and (c) senescent myoblasts labeled with 20 *μ*M DHE and 40 *μ*M carboxy-H_2_DCFDA to detect the presence of ROS which were observed under fluorescence microscope (magnification 200x, scale bar, 200 *μ*m). TRF and ATF diminished the presence of ROS in senescent cells. (d) Quantitative analysis of ROS generation in myoblasts. The percentage of positively stained cells was significantly increased in presenescent and senescent myoblasts; however, the percentage was decreased in TRF-, ATF-, and TRF + NAC-treated senescent myoblasts. (e) A gradual decrease in cell viability with increasing concentrations of H_2_O_2_ was observed, indicating the vital role of redox balance maintenance in cells. ^a^Denoting *p* < 0.05, significantly different compared to young control; ^b^*p* < 0.05, significantly different compared to presenescent myoblasts; ^c^*p* < 0.05, significantly different compared to senescent myoblasts; and ^*∗*#*¥*^*p* < 0.05, significantly different compared to 0 mM. Data are presented as the mean ± SD, *n* = 3.

To elucidate the effects of aging on the oxidative status of myoblasts, we expanded cells in culture until replicative senescence was achieved. The generation of ROS was observed at all stages of cell culture using carboxy-H_2_DCFDA (in green, H_2_O_2_, peroxynitrite, hydroxyl radicals, etc.) and DHE (in orange, superoxide anion) (Figures 2(a)–2(c)). The presence of carboxy-H_2_DCFDA gradually increased in untreated control of young to senescent myoblasts (Figures 2(a)–2(c)). However, similar observations were not observed with DHE. Quantitative analysis showed that the amount of intracellular ROS was significantly increased in presenescent and senescent myoblasts compared to young myoblasts (*p* < 0.05), and senescent myoblasts had the highest levels of ROS, suggesting that senescent cells experience more severe oxidant insults compared to young and presenescent cells (Figure 2(d)).

Both TRF and ATF treatments reduced the amount of intracellular ROS in senescent myoblasts, which was indicated by the decline in fluorescence intensity as well as the total number of positively stained cells (Figure 2(c)). The treatments were likely to diminish ROS in young and presenescent cells as well (Figures 2(a) and 2(b)). Quantitative analysis showed that both TRF and ATF significantly diminished intracellular H_2_O_2_ generation in presenescent and senescent myoblasts (*p* < 0.05) (Figure 2(d)). However, only ATF-treated senescent myoblasts had significantly lower intracellular superoxide anion levels compared to untreated senescent control cells (*p* < 0.05) (Figure 2(d)). Presenescent cells treated with TRF + NAC and ATF + NAC exhibited significantly lower ROS generation (*p* < 0.05) (Figure 2(d)). In senescent myoblasts only, TRF + NAC treatment caused a significant decrease in ROS generation (*p* < 0.05) (Figure 2(d)). A similar reduction in free radical generation was not observed in cells treated with NAC alone, indicating that the antioxidant effects observed were the result of TRF and ATF actions. The presence of ROS in all treatment groups in young, presenescent, and senescent myoblasts can be visualized in Figures 2(a), 2(b), and 2(c), respectively.

To confirm the effects of ROS on normal myoblasts, we measured the cell viability immediately after a short-term of H_2_O_2_ insult. Our results showed decreased cell viability at all stages of aging after 45 min of incubation with 1 mM, 1.5 mM, 2 mM, and 2.5 mM H_2_O_2_, *p* < 0.05 (Figure 2(e)). The steady decline in cell viability with increasing H_2_O_2_ concentration exposure may indicate the vital role of a maintained redox balance in muscle cells.

### 3.3. Effects of Antioxidants on Lipid Peroxidation during Replicative Senescence

**Figure 3:**
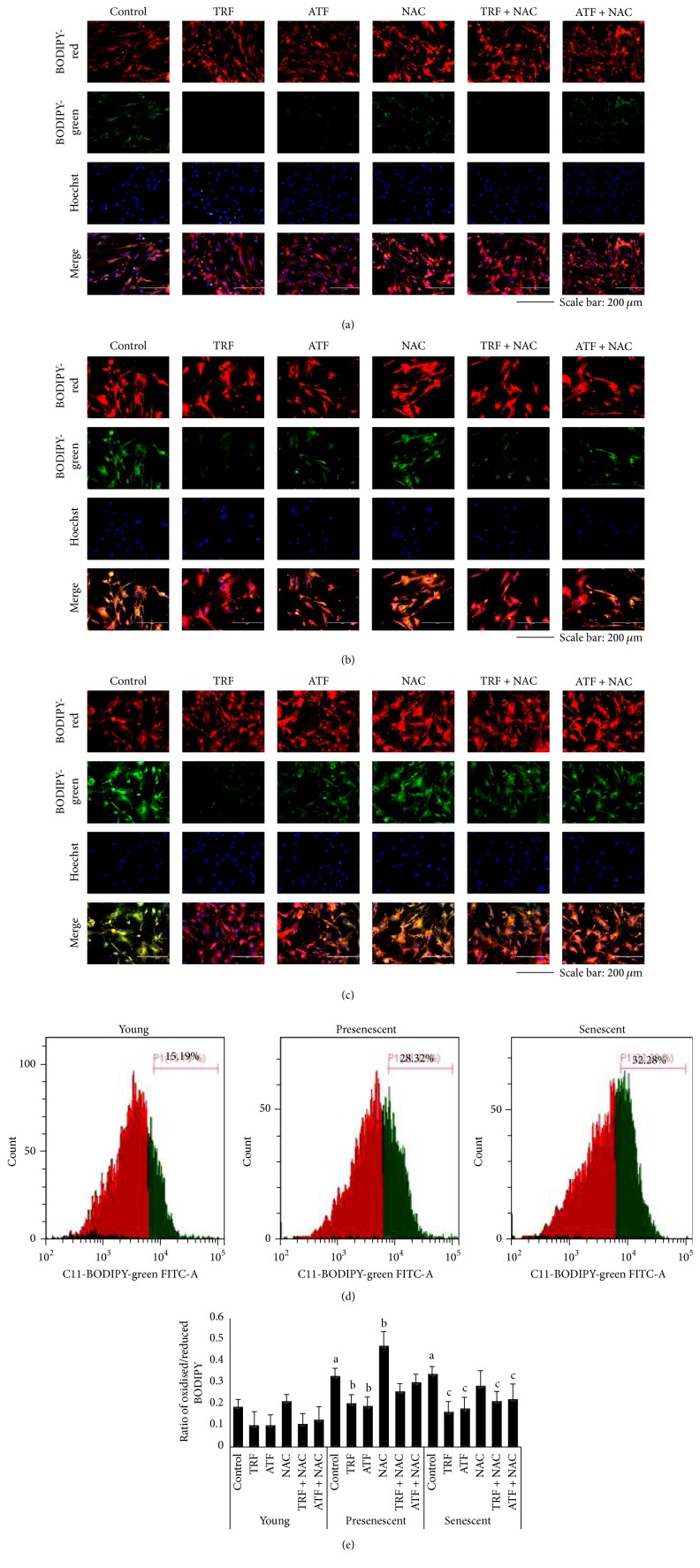
Effects of antioxidants treatment on lipid peroxidation levels of myoblasts. (a) Young, (b) presenescent, and (c) senescent myoblasts labeled with C11-BODIPY® (581/591) to detect lipid peroxidation which were observed under fluorescence microscope (magnification: 200x, scale bar, 200 *μ*m). TRF and ATF treatment effectively reduced lipid peroxidation. (d) Representative histogram of myoblasts based on the oxidized BODIPY (in green, 525/40 bandpass channel). (e) Ratio of oxidized/reduced BODIPY virtually representing lipid peroxidation in myoblast cells. ^a^Denoting *p* < 0.05, significantly different compared to young control; ^b^*p* < 0.05, significantly different compared to presenescent myoblasts; ^c^*p* < 0.05, significantly different compared to senescent myoblasts. Data are presented as the mean ± SD, *n* = 3.

To determine the oxidative damage in myoblasts, lipid peroxidation levels were measured with C11-BODIPY® (581/591), a sensitive fluorescent reporter for lipid peroxidation. The total number of cells that underwent a shift from red to green was gradually augmented from young to senescent cells (Figures 3(a)–3(c)). In senescent myoblasts, the amount of cells with lipid peroxidation, which was indicated by the percentage of cells with oxidized BODIPY (in green), was increased as represented in the right-shifted fluorescence intensity histogram (Figure 3(d)). Quantitative analysis showed that the ratio of cells with oxidized BODIPY to reduced BODIPY (in red) (oxidized/reduced BODIPY ratio) was significantly increased in presenescent and senescent myoblasts compared to young myoblasts (*p* < 0.05) (Figure 3(e)).

Evaluation of lipid peroxidation levels, which was performed after antioxidant treatment, showed that both TRF and ATF reduced the level of lipid peroxidation in young, presenescent, and senescent myoblasts (Figures 3(a), 3(b), 3(c), and 3(e)). The number of oxidized BODIPY-positive cells was decreased in both TRF- and ATF-treated senescent myoblasts (Figure 3(c)). Statistical analysis of the oxidized/reduced BODIPY ratio revealed that both TRF and ATF significantly reduced lipid peroxidation in presenescent and senescent myoblasts (*p* < 0.05) (Figure 3(e)). Surprisingly, NAC increased oxidized/reduced BODIPY ratio in presenescent myoblasts (*p* < 0.05) (Figure 3(e)). However, senescent cells treated with ATF + NAC and TRF + NAC showed significantly decreased lipid peroxidation (oxidized/reduced BODIPY ratio) compared to untreated control senescent myoblasts (*p* < 0.05) (Figure 3(e)).

### 3.4. Cellular Uptake of Vitamin E

**Figure 4:**
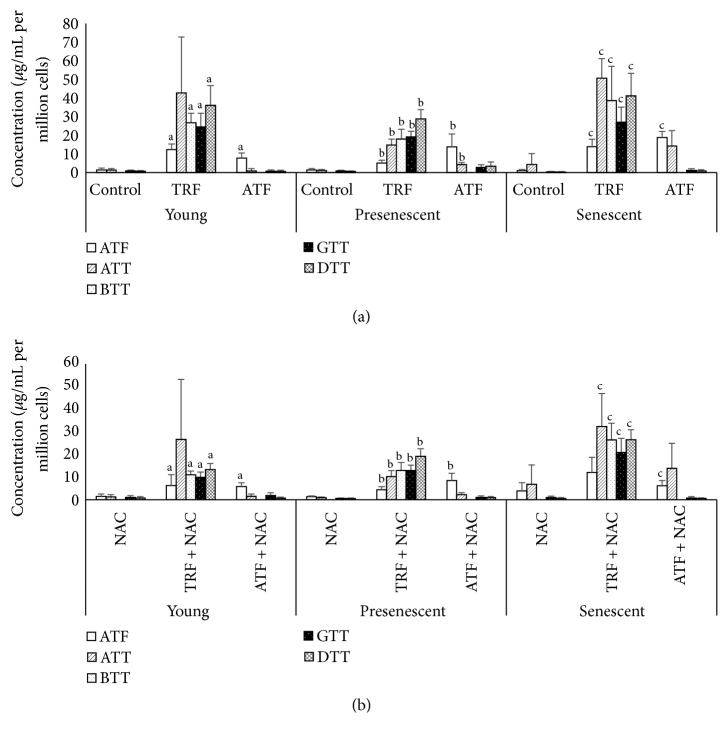
Cellular uptake of vitamin E isomers by myoblasts. (a) Untreated control, TRF- and ATF-treated young, presenescent, and senescent myoblasts. Cellular uptake of 5 vitamin E isomers was significantly higher in TRF-treated myoblasts. (b) NAC-, TRF + NAC-, and ATF + NAC-treated young, presenescent, and senescent myoblasts. Cellular uptake of 5 vitamin E isomers was significantly higher in TRF + NAC-treated myoblasts. ^a^Denoting *p* < 0.05, significantly different compared to the young control, ^b^*p* < 0.05, significantly different compared to the presenescent control, and ^c^*p* < 0.05, significantly different compared to the senescent control. Data are presented as the mean ± SD, *n* = 3.

To validate the cellular uptake of vitamin E, we determined the concentration of vitamin E isomers (ATF, ATT, BTT, GTT, and DTT) in all groups of cells (Figures 4(a) and 4(b)). Young, presenescent, and senescent TRF-treated myoblasts showed the presence of 5 vitamin E isomers, while a significantly inferior number of isomers were found in control cells (Figure 4(a)). A significantly higher concentration of ATF was observed in young, presenescent, and senescent ATF-treated myoblasts compared to untreated cells (*p* < 0.05) (Figure 4(a)). The NAC-treated cells contained the lowest concentrations of vitamin E isomers compared to its combination with TRF or ATF group (Figure 4(b)). TRF + NAC-treated cells contained high levels of all 5 isomers of vitamin E, while ATF + NAC cells only contained a high ATF concentration (Figure 4(b)).

### 3.5. TRF Treatment Modulates Antioxidant Capacity

**Figure 5:**
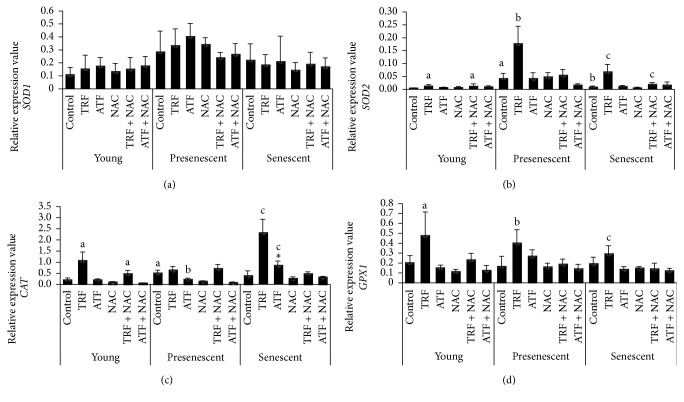
Effects of antioxidants treatment on antioxidant enzymes mRNA expression in young, presenescent, and senescent myoblasts. (a)* SOD1* mRNA, (b)* SOD2* mRNA, (c)* CAT* mRNA, and (d)* GPX1* mRNA expression. ^a^Denoting *p* < 0.05, significantly different compared to the young control, ^b^*p* < 0.05, significantly different compared to the presenescent control, ^c^*p* < 0.05, significantly different compared to the senescent control, and ^*∗*^*p* < 0.05, significantly different compared to TRF-treated senescent myoblasts. Data are presented as the mean ± SD, *n* = 3.

**Figure 6:**
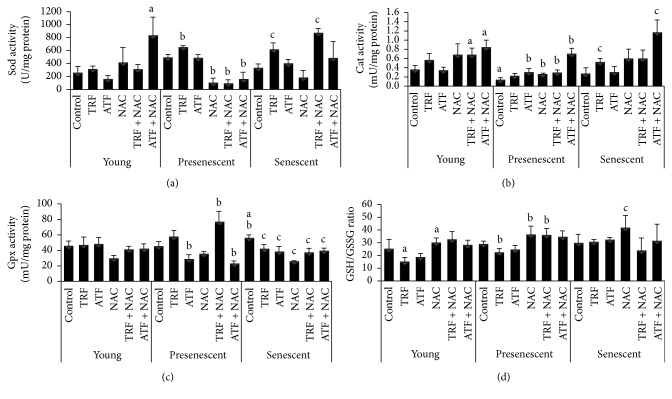
Effects of antioxidants treatment on antioxidant enzymes activities and GSH/GSSG ratio in young, presenescent, and senescent myoblasts. (a) Sod enzyme activity, (b) Cat enzyme activity, (c) Gpx enzyme activity, and (d) GSH/GSSG ratio. ^a^Denoting *p* < 0.05, significantly different compared to the young control, ^b^*p* < 0.05, significantly different compared to the presenescent control, and ^c^*p* < 0.05, significantly different compared to the senescent control. Data are presented as the mean ± SD, *n* = 3.

The modulation of antioxidant capacity by TRF, ATF, NAC, TRF + NAC, and ATF + NAC during the replicative senescence of myoblasts was investigated by determining the mRNA expression of antioxidant enzymes* (SOD1, SOD2, CAT, *and* GPX1)* and activities of antioxidant enzymes (Sod, Cat, and Gpx), as well as the ratio of GSH/GSSG in young, presenescent, and senescent myoblasts (Figures 5 and 6). Overall, TRF treatment was more effective in modulating antioxidant enzymes expression, especially at transcriptional level, compared to other antioxidants in senescent myoblasts.

There was no significant change in* SOD1* mRNA expression levels with aging and antioxidant treatment (Figure 5(a)). However,* SOD2* mRNA expression in the presenescent control was significantly increased compared to the young control (*p* < 0.05) (Figure 5(b)). TRF treatment upregulated* SOD2* mRNA expression in young, presenescent, and senescent myoblasts compared to their corresponding untreated controls (*p* < 0.05) (Figure 5(b)). Furthermore, our results showed that only TRF + NAC modulated the expression of* SOD2* mRNA, while NAC and ATF + NAC did not modulate any antioxidant enzymes at the transcriptional level (Figure 5(b)), suggesting that TRF exerted a modulatory effect at the transcriptional level. Moreover, Sod activity was increased in TRF-treated presenescent and senescent myoblasts compared to their untreated controls (*p* < 0.05) (Figure 6(a)). In senescent myoblasts, treatment with TRF + NAC significantly increased the activity of Sod compared to the senescent control (*p* < 0.05). However, similar increases in Sod activity were observed in young myoblasts treated with ATF + NAC, while, in presenescent myoblasts, treatment with NAC, TRF + NAC, and ATF + NAC decreased Sod activity.

In the presenescent control,* CAT* mRNA expression was significantly higher than the young control, *p* < 0.05 (Figure 5(c)). In contrast, Cat activity in the presenescent control was lower than in the young control (*p* < 0.05) (Figure 6(b)). Treatment with either TRF or ATF upregulated* CAT* mRNA expression in senescent myoblasts compared to untreated controls (*p* < 0.05) (Figure 5(c)), while only TRF increased Cat activity in senescent myoblasts and ATF increased Cat activity in presenescent myoblasts (Figure 6(b)). TRF + NAC significantly increased* CAT* mRNA expression in young myoblasts, in comparison to their untreated controls (Figure 5(c)). Treatment with NAC, TRF + NAC, and ATF + NAC increased Cat activity in presenescent myoblasts (*p* < 0.05), while, in young myoblasts, Cat activity was significantly increased with TRF + NAC and ATF + NAC treatment (Figure 6(b)). However, only ATF + NAC-treated senescent myoblasts demonstrated increased Cat activity.

No significant changes were observed in* GPX1* mRNA expression with aging in myoblasts, even though expression of this gene was modulated by TRF, which was evident by the upregulation of its mRNA expression in young, presenescent, and senescent cells (*p* < 0.05) (Figure 5(d)). However, a similar pattern of expression was not observed for the enzyme activity of Gpx. Gpx activity was significantly higher in the senescent control compared to both the young and presenescent controls (*p* < 0.05) (Figure 6(c)), while both TRF- and ATF-treated senescent myoblasts exhibited lower enzyme activities compared to the senescent control (*p* < 0.05) (Figure 6(c)). Gpx activity levels were increased in TRF + NAC-treated presenescent myoblasts but decreased in ATF + NAC-treated presenescent myoblasts (Figure 6(c)). NAC-, TRF + NAC-, and ATF + NAC-treated senescent myoblasts exhibited significantly lower Gpx activity compared to the untreated senescent control. A similar decrease in Gpx activity was observed in young myoblasts treated with NAC alone.

The GSH/GSSG ratio is always used as an indicator for antioxidant capacity. However, in our study, there was no significant change observed in the GSH/GSSG ratio between young, presenescent, and the senescent control (Figure 6(d)). In young and presenescent myoblasts, TRF treatment reduced the ratio significantly compared to untreated controls (*p* < 0.05) (Figure 6(d)). Determination of the GSH/GSSG ratio in myoblasts showed that treatment with NAC alone in young, presenescent, and senescent myoblasts resulted in a significantly increased GSH/GSSG ratio (*p* < 0.05) (Figure 6(d)). A similar increase in the GSH/GSSG ratio was observed in presenescent cells treated with TRF + NAC.

### 3.6. Effects of Antioxidants on Cell Viability and Apoptosis Profile

**Figure 7:**
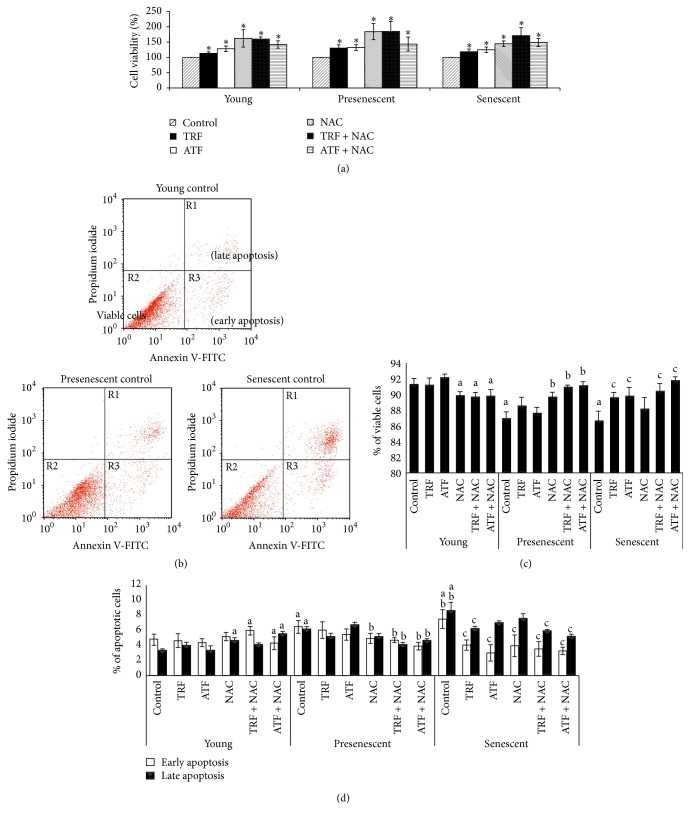
Effects of antioxidants treatment on cell viability and apoptosis. (a) The total number of viable cells after antioxidant treatment of young, presenescent, and senescent myoblasts. (b) Dot plot of Annexin V-FITC and PI double staining. Each quadrant represents different cell conditions, which are late apoptotic cells (FITC^+ve^/PI^+ve^; right upper quadrant; R1); viable cells (FITC^−ve^/PI^−ve^; left lower quadrant; R2); and early apoptotic cells (FITC^+ve^/PI^−ve^; right lower quadrant, R3). The cells shifted from the left lower quadrant to the right lower and upper quadrants when transitioning from young to senescent status. (c) The percentage of viable cells. (d) The apoptosis profile of myoblasts in response to all treatments during early and late apoptosis events. ^*∗*^Denoting *p* < 0.05, significantly different compared to respective control groups, ^a^*p* < 0.05, significantly different compared to young control, ^b^*p* < 0.05, significantly different compared to the presenescent control, and ^c^*p* < 0.05, significantly different compared to the senescent control. Data are presented as the mean ± SD, *n* = 3.

To evaluate the beneficial effects of TRF, ATF, NAC, and their combinations on oxidative status and its final outcome, cell viability (Figure 7(a)) and apoptosis profile (Figures 7(b)–7(d)) were measured. Our results showed that TRF, ATF, NAC, and their combinations significantly increased the number of viable cells in young, presenescent, and senescent myoblasts compared to their corresponding untreated controls (Figure 7(a)).

Apoptotic changes were determined by Annexin V-FITC staining.  Figure 7(b) shows the dot plot of FITC-Annexin V/PI double staining as determined by flow cytometry analysis. The three quadrants represent different cell conditions: the upper right quadrant (R1) indicates nonviable and late apoptotic cells (FITC^+ve^/PI^+ve^), the lower left quadrant (R2) indicates viable cells (FITC^−ve^/PI^−ve^), and the lower right quadrant (R3) indicates early apoptotic cells (FITC^+ve^/PI^−ve^), which is demonstrated by Annexin V binding and cytoplasmic membrane integrity. The quantitative data for the viable cells is shown in Figure 7(c). The percentage of viable cells in the presenescent and senescent controls was significantly lower than in the young control (*p* < 0.05). Treatment with TRF, ATF, TRF + NAC, or ATF + NAC was able to increase the number of viable cells during replicative senescence, signifying the protective role of antioxidant treatment against cell death. A similar increase in cell viability was observed in presenescent myoblasts treated with NAC, TRF + NAC, or ATF + NAC (*p* < 0.05).

Increases in early and late apoptotic events were observed in senescent myoblasts compared to young and presenescent cells (*p* < 0.05) (Figure 7(d)). Treatment with TRF, ATF, NAC, TRF + NAC, or ATF + NAC significantly reduced the number of early apoptotic cells in senescent myoblasts compared to untreated controls (*p* < 0.05). Only TRF, TRF + NAC, and ATF + NAC treatment significantly decreased the number of cells undergoing late apoptotic events (*p* < 0.05). Early and late apoptotic events were also increased in presenescent myoblasts and were reduced by TRF + NAC or ATF + NAC treatment (*p* < 0.05).

## 4. Discussion

Vitamin E is well known for its free radical-scavenging capacity, which plays an important role in antioxidant defense mechanisms. The lesser known form of vitamin E, tocotrienols, has been reported to possess greater antioxidant effects and better membrane penetration ability compared to tocopherols [26, 27]. These features contribute to their efficient uptake by targeted cells in addition to exhibiting higher scavenging power due to active recycling [26, 28]. The present study has demonstrated the effectiveness of TRF, a broad mixture of vitamin E, in combating oxidative stress and enhancing antioxidant defense mechanisms in senescent myoblasts, resulting in the improvement of senescence-associated oxidative stress as evidenced by decreased programmed cell death and increased cell viability.

In brief, increased oxidative stress as a result of redox imbalance during aging leads to increased susceptibility of satellite cells to apoptosis and affects muscle regeneration [6, 8, 29, 30]. The findings of this study showed that ROS generation was significantly increased in senescent myoblasts, resulting in higher levels of oxidative damage as indicated by elevated lipid peroxidation. Increased oxidative stress during replicative senescence has been reported to be similar to the conditions in satellite cells derived from aged individuals [8, 10]. Moreover, elevated ROS in myoblasts endangers cellular endurance as shown in our study. We found that the number of viable cells decreased with increasing exogenous H_2_O_2_ concentration [31].

The presence of free radicals in cells can be counteracted by antioxidant defense systems. However, antioxidant capacity decreases with advancing age, resulting in the accumulation of free radicals, which threaten cell viability [8]. As reported by Fulle and his coworkers [8], the levels of the antioxidant enzymes Cat and glutathione transferase in satellite cells isolated from the elderly were drastically decreased compared to cells from young donors.* SOD1* and* GPX1 *mRNA expression were not significantly different across the cell stages. However,* SOD2 *and* CAT* mRNA expression were upregulated in presenescent myoblasts, which was similar to Sod activity in a previous study [32]. Additionally, a decline in Cat activity was observed in presenescent myoblasts compared to young myoblasts. Because antioxidant enzymes are regulated in response to oxidative stress [33], we postulated that presenescent myoblasts were attempting to compensate for the decreased Cat levels by upregulating* CAT* mRNA expression to overcome the progressive increase in oxidative stress. Previous study reported that there is a compensatory machinery in antioxidant defense systems which maintains the integrity of muscle [34]. However, we found that senescent cells were less responsive to increased oxidative stress. The levels of* SOD2* mRNA in senescent cells were lower compared to presenescent cells. The expression of* SOD2* mRNA was critical because lack of gene* SOD2* can cause mitochondrial damage and lifespan shortening in* Drosophila* [35]. On the other hand, Gpx activity was increased in senescent myoblasts compared to young and presenescent myoblasts. A previous study also reported enhanced Cat and Gpx activity during aging, which could be an adaptive mechanism to the elevated H_2_O_2_ levels [36], but the response may be inadequate to counteract the existing ROS.

Our study also reports on the nonenzymatic antioxidants in senescent myoblasts, glutathione [9]. The GSH/GSSG ratio in our study remained unchanged at all ages, which is similar to data reported previously, suggesting that there is no alteration in GSH membrane transportation during aging [37]. Therefore, based on the intracellular ROS levels, lipid peroxidation, and enzymatic antioxidants, our results indicated that senescent myoblasts experience oxidant insults as a result of a less effective antioxidant defense system, which barely counteracted the elevated levels of cellular senescence-associated oxidative stress.

The vitamin E concentration in cells increased with vitamin E treatment (TRF or ATF alone), particularly in TRF-treated myoblasts, which contained all five isomers that were tested. NAC treatment did not affect vitamin E uptake by the cells, as shown by higher levels of vitamin E in myoblasts treated with TRF + NAC and ATF + NAC compared to cells treated with NAC alone. In brief, vitamin E can act as a nonenzymatic antioxidant to combat oxidative stress [9]. Thus, given its free radical-scavenging power, both TRF-treated and ATF-treated senescent myoblasts demonstrated reductions in their intracellular ROS generation and lipid peroxidation levels. Findings from a previous animal study reported a similar reduction in lipid peroxidation levels after TRF and ATF supplementation [38], revealing the protective effects of vitamin E against oxidative damage. TRF has been reported to improve senescence-associated phenotypes in H_2_O_2_-induced myoblasts [18]. In another study, ATF showed protective effects in H_2_O_2_-induced myoblasts [39]. Therefore, both TRF and ATF could potentially be used to protect cells against reactive oxidants.

Our data showed that TRF regulates the expression of antioxidant enzymes in young, presenescent, and senescent myoblasts.* SOD2 *mRNA expression was upregulated in all TRF-treated cells, while Sod enzyme activity was upregulated in TRF-treated presenescent and senescent myoblasts. The* SOD2 *mRNA encodes one of the Sod isoforms (MnSod), which is located in mitochondrial matrix [40]. Gianni et al. [41] suggested that a superoxide related mitochondrial stress is more apparent than cytosolic stress during aging. Previous studies also reported that increased mitochondrial DNA or RNA mutations were correlated with increasing age and abnormality of aged muscle [42, 43]. Hence, age-related oxidative stress is thought to lead to mitochondrial dysfunction, which eventually may lead to progressive loss of muscle mass and strength [44]. Instead of* SOD1* mRNA, TRF regulated* SOD2* mRNA; thus, we hypothesized that TRF may act on the mitochondria and potentially protect this organelle during aging. The upregulation of* SOD2* mRNA by TRF could be a compensatory mechanism to counteract elevated oxidative challenges in mitochondria during replicative senescence and prevent accumulation of oxidative damage that can trigger cellular aberration. Evidence demonstrated that *γ*-tocotrienol potentially protects renal proximal tubular cells from oxidant-induced mitochondria dysfunctional and cellular injury [45]. Increased Sod activity with TRF treatment further validated the ability of TRF to modulate the decomposition of superoxide anions to H_2_O_2_ [38]. However, neither* Sod* mRNA nor enzyme activity was modulated by ATF.

In addition to the significant regulation of Sod activity, TRF treatment also upregulated* CAT* and* GPX1 *mRNA in young and senescent myoblasts. However, at the enzyme activity level, TRF increased Cat activity, whereas Gpx activity was reduced in senescent myoblasts. Cat and Gpx work in parallel to remove H_2_O_2_ in cells [9]. Gpx also catalyzes the elimination of hydroperoxides originated from unsaturated fatty acids at the expense of reduced glutathione [46]. However, the protective role of the glutathione redox cycle is limited at low levels of oxidative stress. During severe oxidant insults, Cat becomes more substantial [46]. Thus, increased Cat at the mRNA and enzyme activity revealed the effectiveness of TRF in enhancing the antioxidant defense system in senescent myoblasts. Because Gpx is involved in hydroperoxide degradation, decreased Gpx in TRF-treated senescent myoblasts may be attributable to decreased lipid peroxidation in cells as reported in this study. This explanation can also be applied to ATF-treated myoblasts, which exhibited decreased Gpx enzyme activity in both the presenescent and senescent stages. In addition, ATF treatment upregulated* CAT* mRNA in senescent cells but at levels that were significantly lower than TRF-treated cells. These findings indicate that TRF potentially improves the antioxidant defense system in senescent myoblasts, and this effect is better than that of ATF.

NAC is a precursor of cysteine that can sustain the production of glutathione, an important antioxidant in cells [47]. In this study, NAC treatment successfully increased the GSH/GSSG ratio in all myoblasts. NAC can scavenge ROS directly [47]. In a previous study, NAC showed protective effects against dystrophic muscle damage in the* mdx* mouse [48] and attenuates fatigue during prolonged exercise [49]. Our results showed that NAC ameliorated myoblast morphology and SA-*β*-gal staining during senescence, which is similar to the effects of TRF and ATF [19]. Similar effects were also observed in cells treated with combinations of TRF + NAC and ATF + NAC. However, intracellular ROS and lipid peroxidation levels were not modulated by NAC treatment alone during senescence. The rate constants of NAC in reaction with superoxide anion, H_2_O_2_, and peroxynitrite were comparatively low [47]; consequently, the influence of NAC on the dyes used in this study would be limited. In our study, lipid peroxidation levels were not improved by NAC, as supported by a previous study that measured malondialdehyde (MDA) as a product of lipid peroxidation in the* mdx* mouse [48]. As expected, both combined treatment with TRF + NAC and ATF + NAC lowered lipid peroxidation levels. On the other hand, intracellular ROS levels were only ameliorated by TRF + NAC treatment in senescent myoblasts, suggesting that TRF is better than ATF in scavenging ROS, even at lower concentrations, compared to the concentration used for TRF treatment alone.

The effects of NAC on antioxidant enzymes were only observed for Gpx activity in senescent myoblasts, although a previous study showed that NAC was able to increase antioxidant enzymes in cocaine-induced hepatocytes [50]. We found that NAC modulated the antioxidant enzyme activity in presenescent myoblasts as shown by decreased Sod activity and increased Cat activity with NAC treatment. Treatment of senescent myoblasts with the combination of TRF + NAC resulted in a stronger antioxidant defense response, which involves the upregulation of* SOD2* mRNA expression and the increment in Sod activity, as well as a decrease in Gpx activity, compared to treatment with NAC alone. These results were similar to those observed for TRF-treated senescent myoblasts. Regarding ATF, the combination of ATF + NAC exerted greater effects on antioxidant enzymes compared to treatment with ATF alone. Both Sod activity and Gpx activity decreased whereas Cat activity increased in presenescent myoblasts with ATF + NAC treatment. Increased Cat activity and decreased Gpx activity were also observed in ATF + NAC-treated senescent myoblasts. From the results, both TRF + NAC and ATF + NAC treatment were more effective than NAC alone in ameliorating the antioxidant defense system, which is similar to previous study that showed combination of NAC and vitamin E has better effect on gentamycin-induced nephrotoxicity compared to NAC alone [51]. Therefore, the improved antioxidant defense mechanisms observed in this study may be attributable to the modulatory effects of vitamin E treatment.

Satellite cells play very important roles in regenerating injured muscle fibers [1]. Despite the availability of satellite cells, which decline with increasing age [52, 53], they may not be the sole reason underlying the impaired regenerative response to injury during aging. Decreased satellite cell proliferative capacity was reported with increased susceptibility to apoptosis, signifying the fact that programmed cell death may also play a part in age-related muscle regeneration impairment [29]. Under stressful stimuli, more satellite cells in old animals underwent apoptosis, thereby distorting skeletal muscle regeneration [29]. However, in a previous study, vitamin E was reported to be able to protect against cell death induced by low doses of oxidants [39]. In another study, it was reported that antioxidant levels were interrelated with the regenerative capacity of muscle stem cells [30]. In short, viable senescent myoblasts were preserved, and the amount of cell death was diminished with TRF treatment, which may be attributed to the improvement of the oxidative status in senescent cells. This may indicate that TRF ameliorates regenerative capacity during aging in human myoblasts [19].

Adequate supply of vitamin E is essential for muscle health. Conversely, vitamin E deficiency can lead to poor muscle performance [12, 14]. A study showed that the antioxidant properties alone are insufficient to repair the injured myoblasts [17]. Lipid-soluble vitamin E can easily diffuse into the hydrophobic membrane and act as a “stabilizer” for lipid membranes to facilitate ROS scavenging activity [17]. Accordingly, the findings of our study showed that both TRF and ATF produced greater effects than NAC in oxidative damage prevention. However, ATF, which is a well-known representative of vitamin E, was not as effective as TRF in protecting against replicative senescence in myoblasts. Although ATF improved the oxidative damage to a similar degree as TRF, TRF is superior in enhancing antioxidant defense mechanism. Previous findings showed that TRF-treated rats exhibited better physical performance and oxidative status than ATF-treated rats [38]. Unlike ATF, TRF is a broad mixture of vitamin E that contains all four isomers of tocotrienol and ATF; thus it should be more potent in scavenging free radicals. The distinctive antioxidant properties of each isomer have been attributed mainly to their chemical structure [54]. For instance, the unsaturated isoprenoid side chain of tocotrienols accounts for a higher peroxyl radical-scavenging potential compared to tocopherols [54]. As a result, even at a lower concentration, TRF is able to improve antioxidant defense mechanism and ameliorate the oxidative status of senescent myoblasts. Previous report suggested that, at low concentrations, *γ*-tocotrienol can protect cells against H_2_O_2_-induced apoptosis, while a higher concentration is required for ATF to produce the similar outcome [22]. Our results showed the modulatory effect of TRF is distinguishable compared to other treatments. Previous findings reported that tocotrienols can target a broad range of molecules that might play a role in aging or degenerative diseases [55]. Hence, we suggest that TRF is better than ATF in modulating antioxidant enzymes, particularly at the transcriptional level, to reestablish redox balance in senescent myoblasts.

In summary, our study highlights the effects of TRF on oxidative status in myoblasts during replicative senescence. The results of our study showed increased oxidative stress in senescent myoblasts with reduced antioxidant capacity and increased susceptibility towards programmed cell death or apoptosis, which were distinguishable from young myoblasts. Treatment with TRF in senescent myoblasts resulted in diminished ROS and lipid peroxidation in addition to reinforcing the antioxidant defense system by augmenting antioxidant enzymes levels in senescent myoblasts, which ultimately maintained the number of myoblast cells. In conclusion, TRF is a useful antioxidant that can counteract oxidative stress and improve cellular survival during replicative senescence of myoblasts, and thereby, it can potentially be used to ameliorate muscle regeneration such as sarcopenia, although further experiments should be carried out.

## Supplementary Material

The supplementary materials contain the figures which showed the effects of NAC treatment, combination of TRF and NAC and combination of ATF and NAC on the cell viability of young, presenescent and senescent myoblasts (Supplemental 1), and the figures which displayed the melt curve analysis and agarose gel electrophoresis for the primer specificity determination (Supplemental 2).

## Figures and Tables

**Table 1:** The primer sequences of *SOD1*, *SOD2*, *CAT,* and *GPX1 *[21].

Gene	Forward	Reverse	Product size
*SOD1*	GAAGGTGTGGGGAAGCATTA	ACATTGCCCAAGTCTCCAAC	174
*SOD2*	CGTCACCGAGGAGAAGTACC	CTGATTTGGACAAGCAGCAA	313
*CAT*	CGTGCTGAATGAGGAACAGA	AGTCAGGGTGGACCTCAGTG	119
*GPX1*	CCAAGCTCATCACCTGGTCT	TCGATGTCAATGGTCTGGAA	127
*GAPDH*	TCCCTGAGCTGAACGGGAAG	GGAGGAGTGGGTGTCGCTGT	218
