# Supplementary figures and images for: Tocotrienol-Rich Fraction Ameliorates Antioxidant Defense Mechanisms and Improves Replicative Senescence-Associated Oxidative Stress in Human Myoblasts
Source: Oxid Med Cell Longev. 2017 Jan 24;2017:3868305. doi: 10.1155/2017/3868305 (PMC5294752; doi:10.1155/2017/3868305)

Supplementary 1


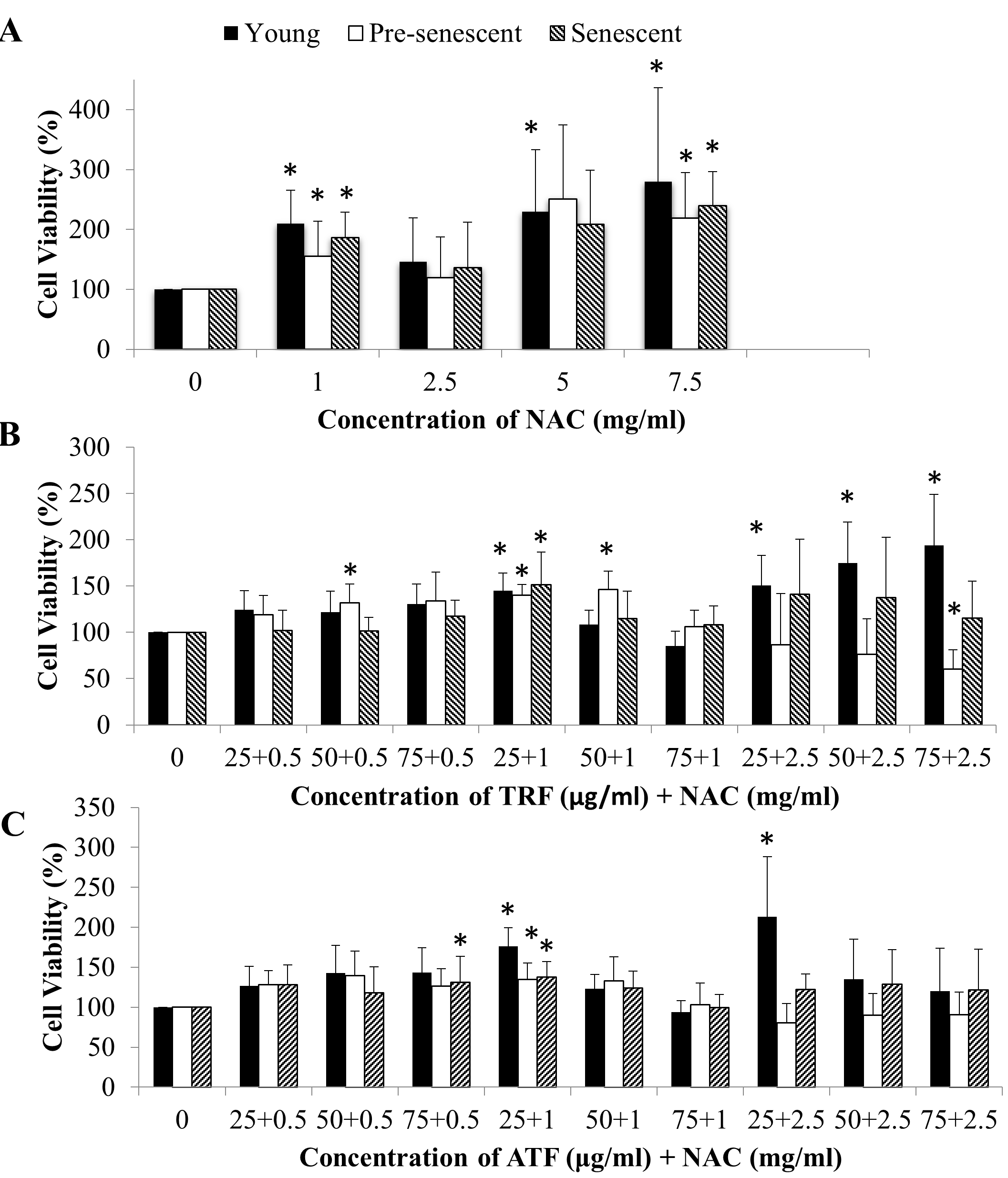


Supplementary 2


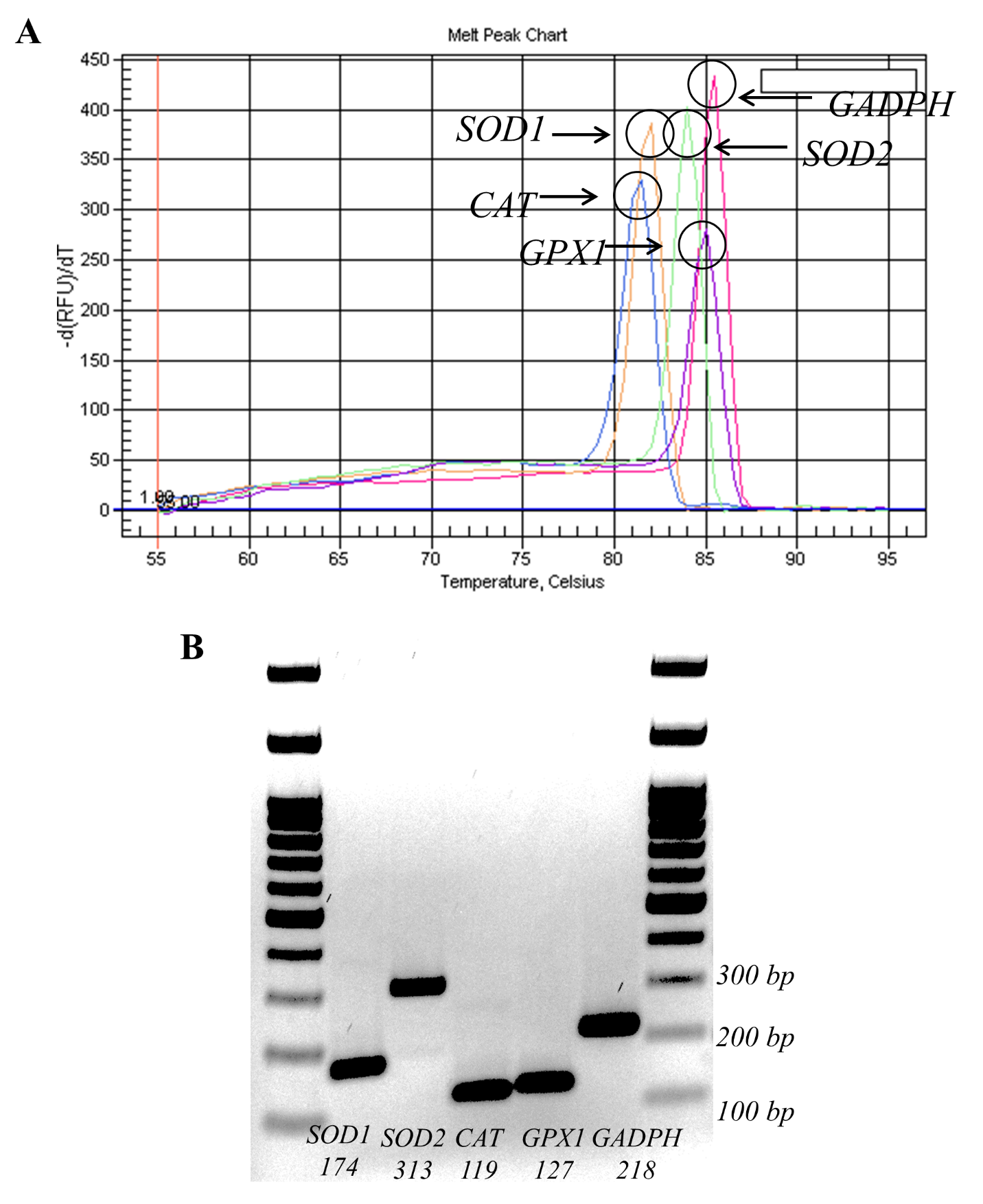

Supplement: Supplementary file 1 — The supplementary materials contain the figures which showed the effects of NAC treatment, combination of TRF and NAC and combination of ATF and NAC on the cell viability of young, presenescent and senescent myoblasts (Supplemental 1), and the figures which displayed the melt curve analysis and agarose gel electrophoresis for the primer specificity determination (Supplemental 2). [file 3868305.f1.docx]
